# SHMT2 Promotes Gastric Cancer Development through Regulation of HIF1α/VEGF/STAT3 Signaling

**DOI:** 10.3390/ijms24087150

**Published:** 2023-04-12

**Authors:** Weida Wang, Mingjin Wang, Tingting Du, Zhenyan Hou, Shen You, Sen Zhang, Ming Ji, Nina Xue, Xiaoguang Chen

**Affiliations:** 1 Institute of Materia Medica, Chinese Academy of Medical Sciences and Peking Union Medical College, Beijing 100050, China; 2 State Key Laboratory of Bioactive Substance and Function of Natural Medicines, Beijing 100050, China

**Keywords:** gastric cancer, SHMT2, HIF1α, redox balance, progression

## Abstract

The metabolic enzymes involved in one-carbon metabolism are closely associated with tumor progression and could be potential targets for cancer therapy. Recent studies showed that serine hydroxymethyltransferase 2 (SHMT2), a crucial enzyme in the one-carbon metabolic pathway, plays a key role in tumor proliferation and development. However, the precise role and function of SHMT2 in gastric cancer (GC) remain poorly understood. In this study, we presented evidence that SHMT2 was necessary for hypoxia-inducible factor-1α (HIF1α) stability and contributed to GC cells’ hypoxic adaptation. The analysis of datasets retrieved from The Cancer Genome Atlas and the experimentation with human cell lines revealed a marked increase in SHMT2 expression in GC. The SHMT2 knockdown in MGC803, SGC7901, and HGC27 cell lines inhibited cell proliferation, colony formation, invasion, and migration. Notably, SHMT2 depletion disrupted redox homeostasis and caused glycolytic function loss in GC cells under hypoxic circumstances. Mechanistically, we discovered SHMT2 modulated HIF1α stability, which acted as a master regulator of hypoxia-inducible genes under hypoxic conditions. This, in turn, regulated the downstream VEGF and STAT3 pathways. The in vivo xenograft experiments showed that SHMT2 knockdown markedly reduced GC growth. Our results elucidate the novel function of SHMT2 in stabilizing HIF1α under hypoxic conditions, thus providing a potential therapeutic strategy for GC treatment.

## 1. Introduction

Gastric cancer (GC) ranks as the third most common cause of cancer-related deaths and the fifth most prevalent disease worldwide, accounting for 10% of global cancer fatalities. In advanced cases, the median survival time is 10–12 months [1,2]. Due to GC’s heterogeneity, predicting therapy outcomes is challenging. A better understanding of the metabolic characteristics of gastric carcinogenesis could significantly impact the prevention and treatment strategies for patients with the precancerous disease. However, the molecular pathways that trigger GC remain poorly understood. Despite significant developments in surgical treatment, chemotherapy, radiation, tyrosine kinase inhibitors, and immunotherapy over the past few decades, the five-year survival rate for patients with gastric adenocarcinoma is only 57.8% [3,4,5,6]. Cancer metastasis and recurrence are substantially responsible for the poor prognosis. Therefore, it is urgent to find more efficient GC diagnostic and therapeutic targets.

One-carbon metabolism (OCM) is a crucial biochemical pathway that transfers a single carbon unit from one molecule to another, supporting essential biological processes, including DNA synthesis, amino acid metabolism, and epigenetic regulation [7]. However, recent research suggested that disruptions in this pathway may contribute to GC development, with key enzymes such as methylenetetrahydrofolate reductase (MTHFR), methionine synthase (MTR), and methionine synthase reductase (MTRR) [8]. Dietary factors, including folate and vitamin B12, which are vital for OCM, have also been associated with an increased GC risk. Kim et al. conducted a correlation analysis between folate intake and GC risk, finding that a low folate intake can significantly reduce GC risk, possibly due to gene–folate interactions [9]. In a prospective study, Kweon et al. found that a high folate intake was associated with an increased GC risk in premenopausal women [10]. Despite there being an incomplete understanding of the exact mechanisms by which disruptions in OCM lead to GC, the evidence suggests that this pathway plays a crucial role in GC.

Serine hydroxymethyltransferase 2 (SHMT2) is an essential enzyme in the one-carbon metabolic cycle, located in the mitochondria, and responsible for producing the one-carbon units; its isoenzyme, SHMT1, is responsible for the metabolism of serine to glycine in the cytoplasm [11]. Tumor cells can retain the supply of one-carbon units necessary for proliferation by altering these metabolic enzymes. Recent studies have demonstrated that SHMT2 is involved in DNA repair, RNA translation, epigenetic changes, and redox defense [11,12,13]. SHMT2 is significantly upregulated in various cancers, including colorectal cancer, glioma, renal cancer, and bladder cancer [14,15,16,17]. Clinical studies have also shown that high SHMT2 expression is closely related to tumor invasion and prognosis [18]. Therefore, SHMT2 is expected to be an important target for tumor metabolic reprogramming, and further exploration of its role in tumors is needed. SHMT2 is also associated with GC development. Shi’s research revealed that elevated SHMT2 expression facilitated GC progression, and it also functioned as an autonomous prognostic biomarker for GC [19]. Furthermore, SHMT2 reduction improved the sensitivity of GC cells to radiotherapy by regulating the Wnt/β-catenin pathway [20]. These studies suggest that SHMT2 is a potential crucial target in GC treatment.

In this study, we explored the expression, clinical characteristics, and prognostic significance of SHMT2 in GC patients. We also examined the effects of SHMT2 on GC cell proliferation, apoptosis, and migration in vitro, and discovered that SHMT2 is essential for maintaining the basic physiological functions of GC cells under hypoxic conditions. Furthermore, we identified the involvement of SHMT2 in the signaling pathway of hypoxia-inducible factor-1α (HIF1α), VEGF, and STAT3, and validated these findings in vivo. Therefore, our study highlighted the SHMT2’s clinical relevance in GC patients and provided insights into its biological function, potential molecular mechanisms, and regulation in GC cells.

## 2. Results

### 2.1. SHMT2 Was Highly Expressed in Gastric Cancer Patients and Was Accompanied by a Poor Prognosis

**Figure 1:**
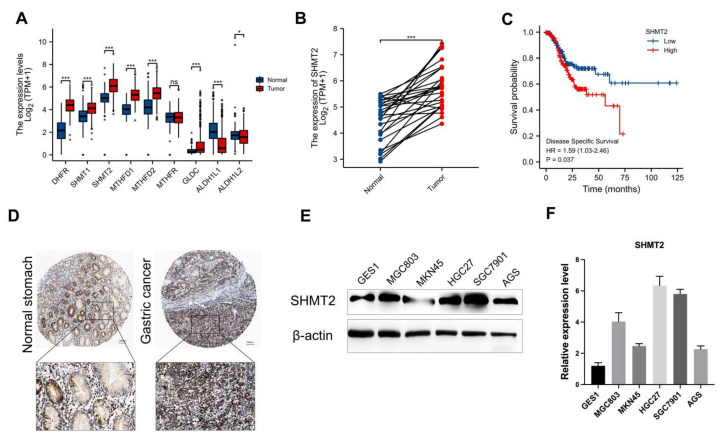
Serine hydroxymethyltransferase 2 (SHMT2) was highly expressed in patients with gastric cancer (GC) and was associated with a poor prognosis. (**A**) Differential expression of genes related to one-carbon metabolism in GC patients based on The Cancer Genome Atlas (TCGA)-GC samples. (**B**) The relative mRNA levels of SHMT2 in 27 pairs of normal gastric and tumor tissues from TCGA-GC samples. (**C**) Prognostic analysis of disease-specific survival in GC patients stratified by SHMT2 expression levels using Kaplan–Meier plots. (**D**) Representative IHC staining of SHMT2 in normal gastric and tumor tissues from GC patients from the Human Protein Atlas. Scale bar, 200 μm and 50 μm. (**E**) Expression of SHMT2 protein level in normal gastric cells and GC cells. (**F**) Expression of SHMT2 mRNA level in normal gastric cells and gastric cancer cells. * *p* < 0.05, *** *p* < 0.001; ns: no significance.

We analyzed data from The Cancer Genome Atlas (TCGA) database and GTEx database of 414 cases of GC patients and 210 corresponding normal tissues. We searched for the expression levels of the mRNAs of the genes related to OCM, including DHFR, MTHFR, SHMT1, SHMT2, MTHFD1, MTHFD2, GLDC, ALDH1L1, and ALDH1L2. We found that the OCM-related genes were highly expressed in the GC patients, indicating that OCM was highly activated in GC (Figure 1A). Among these enzymes, SHMT2, a key enzyme in OCM, had the highest level of expression compared to others. In a paired analysis of 27 cases from each group in the database, the mean value for the normal group was 4.479 ± 0.805 and for the tumor group was 5.761 ± 0.84. A paired samples t-test showed that the tumor group was higher than the normal group, with a statistically significant difference of 1.282 (0.912–1.652) between the two groups (*p* < 0.001) (Figure 1B). We also conducted a disease-specific survival analysis in GC patients with a high SHMT2 expression (156 cases) and a low SHMT2 expression (175 cases) and found that the patients with a high SHMT2 expression had worse prognoses than those with a low expression. The COX regression analysis supported this finding, with a statistically significant difference in survival time distribution for the subgroups (*p* = 0.037) (Figure 1C). We also assessed SHMT2 expression in clinical immunohistochemistry staining of GC and normal gastric tissues using the Human Protein Atlas database. While SHMT2 was expressed in both normal and cancerous tissues, the number of positive stains was significantly higher in the cancerous tissues (Figure 1D). By using Western blot and real-time PCR, we assessed the levels of SHMT2 protein and RNA expression in the human gastric normal cell line GES1 and the human GC cell lines MGC803, MKN45, HGC27, SGC7901, and AGS. The results showed that SHMT2 expression was higher in GC cell lines than in normal gastric cells (Figure 1E,F). Our findings suggest that SHMT2 could be a promising target for GC treatment and have important clinical value.

### 2.2. The Proliferation of Gastric Cancer Cells In Vitro Was Impacted by the Deletion of SHMT2

**Figure 2:**
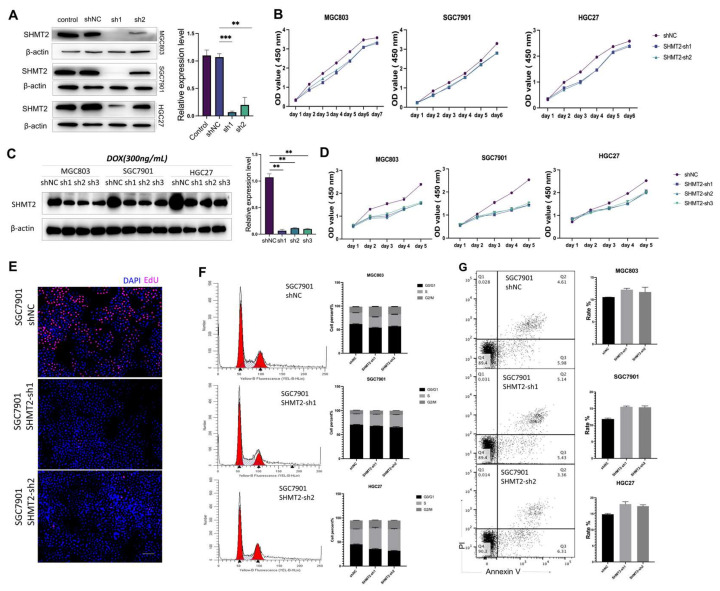
Inhibition of SHMT2 suppressed GC cell proliferation and induced apoptosis. (**A**) Western blot and real-time PCR identified the knockdown efficiency of SHMT2 in MGC803, SGC7901, and HGC27. (**B**) CCK-8 assay was performed to evaluate the proliferation of MGC803, SGC7901, and HGC27 cells after transfection with shSHMT2. (**C**) Western blot and real-time PCR confirmed the SHMT2 knockdown efficiency in MGC803, SGC7901, and HGC27 with doxycycline (DOX) (300 ng/mL) induction. (**D**) CCK-8 assay measured the proliferation of shSHMT2 MGC803, SGC7901, and HGC27 cells after DOX (300 ng/mL) induction. (**E**) EdU assay detected the effect of the proliferation of shSHMT2 MGC803, SGC7901, and HGC27 cells. Scale bar, 100 μm. (**F**) Effect of SHMT2 knockdown on the cell cycle of MGC803, SGC7901, and HGC27 cells. (**G**) Effect of SHMT2 knockdown on apoptosis in MGC803, SGC7901, and HGC27 cells (Q2 + Q3). ** *p* < 0.01, *** *p* < 0.001.

GC cell lines, MGC803, SGC7901, and HGC27, were selected to investigate SHMT2’s role in GC due to their high SHMT2 expression. A stable SHMT2 knockdown cell line was constructed for these cell lines using shRNA. Western blot and real-time PCR were used to confirm the knockdown efficiency (Figure 2A). The CCK-8 assay showed a slight inhibitory effect of SHMT2 deficiency on cell proliferation, but the difference was not significant (Figure 2B). We used a Tet inducible system to transiently silence the SHMT2 gene by adding doxycycline (DOX) to the infected GC cells with pKO.1-Tet-shSHMT2 lentivirus. The knockdown efficiency was confirmed using Western blot and real-time PCR (Figure 2C). However, the DOX-induced transient model of SHMT2 significantly inhibited proliferation (Figure 2D), which may have been due to compensatory mechanisms in the stable transient cells. The EdU labeling of proliferating SGC7901 cells showed that the SHMT2 knockdown reduced cell proliferation (Figure 2E). Flow cytometry analysis showed that the SHMT2 knockdown slightly increased the proportion of cells in the S-phase, which inhibited cellular DNA synthesis (Figure 2F). Additionally, SHMT2 knockdown induced apoptosis, but the effect was not significant (Figure 2G). We also tested SHIN1, an inhibitor of SHMT2, in the three GC cell lines. The results showed that SHIN1 had a limited ability to kill the cells, with IC50s of 2.31 μM, 2.59 μM, and 2.22 μM, respectively, which were consistent with the knockdown results (Figure S1). We also used SHIN1 for the cell cycle and apoptosis assays (Figures S2 and S3) and the results were consistent with the knockdown results. The above findings indicated that SHMT2 inhibition had some effect on GC cell proliferation and growth, but not to a statistically significant extent.

### 2.3. SHMT2 Deletion Affected the Ability of Gastric Cancer Cells to Invade and Metastasize

**Figure 3:**
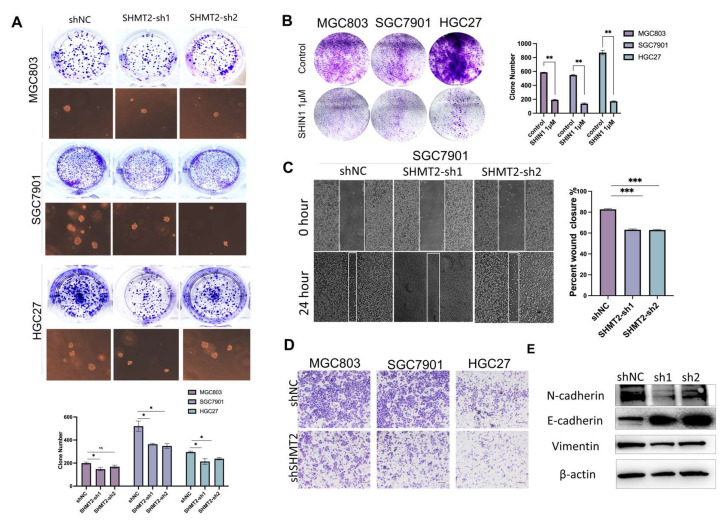
SHMT2 knockdown inhibited GC cell invasion and metastasis. (**A**) Representative images of SHMT2 knockdown’s effect on cell colony formation (plate clones and soft agar clones) and quantitative analysis of colony formation. (**B**) Representative images and quantitative analysis of the effect of SHIN1 (1 μM) on cell colony formation. (**C**) Transwell assay detected the effect of SHMT2 knockdown on the cell migration ability and the representative images. Scale bar, 100 μm. (**D**) Representative images and quantitative analysis of SHMT2 knockdown’s effect on the cell migration ability detected using a 24 h wound-healing assay in SGC7901. Scale bar, 100 μm. (**E**) Western blot examined SHMT2 knockdown’s effect on the expression of cellular EMT markers. * *p* < 0.05, ** *p* < 0.01, and *** *p* < 0.001; ns: no significance.

Tumor invasion and metastasis are characteristics in addition to proliferation. We investigated SHMT2 knockdown’s impact on invasive metastasis in vitro. First, we observed a significant decrease in cell colony formation in MGC803, SGC7901, and HGC27 cells following SHMT2 knockdown (Figure 3A). Furthermore, inhibition of SHMT2 via pharmacological means also hindered clonogenesis in a clone-forming assay (Figure 3B). We performed a wound healing assay to examine the healing area of the SGC7901 cells after 24 h, and observed a slower healing rate in the shSHMT2 group compared to the shNC group (Figure 3C). In addition, the transwell assay revealed a significant decrease in the migration ability of cells after SHMT2 knockdown (Figure 3D). These results indicated that SHMT2 knockdown significantly impaired the migration and invasion ability of the MGC803, SGC7901, and HGC27 cells. Previous studies have well-established a significant association between the epithelial–mesenchymal transition (EMT) process and cell invasion in cancer development [21]. To explore whether the reduced migration ability of GC cells was associated with EMT, we performed a Western blot analysis to assess the expression of EMT–related markers, including N-cadherin, E-cadherin, and vimentin in SGC7901. The SHMT2 knockdown resulted in significantly lower levels of N-cadherin and vimentin and higher levels of E-cadherin in GC cells (Figure 3E). These findings suggested that SHMT2 may play a role in GC progression.

### 2.4. SHMT2 Deficiency Affected Cell Proliferation, Cell Cycle, Apoptosis, Invasion, and Metastasis, Together with a Major Impact on Glycolysis in Hypoxic Environments

**Figure 4:**
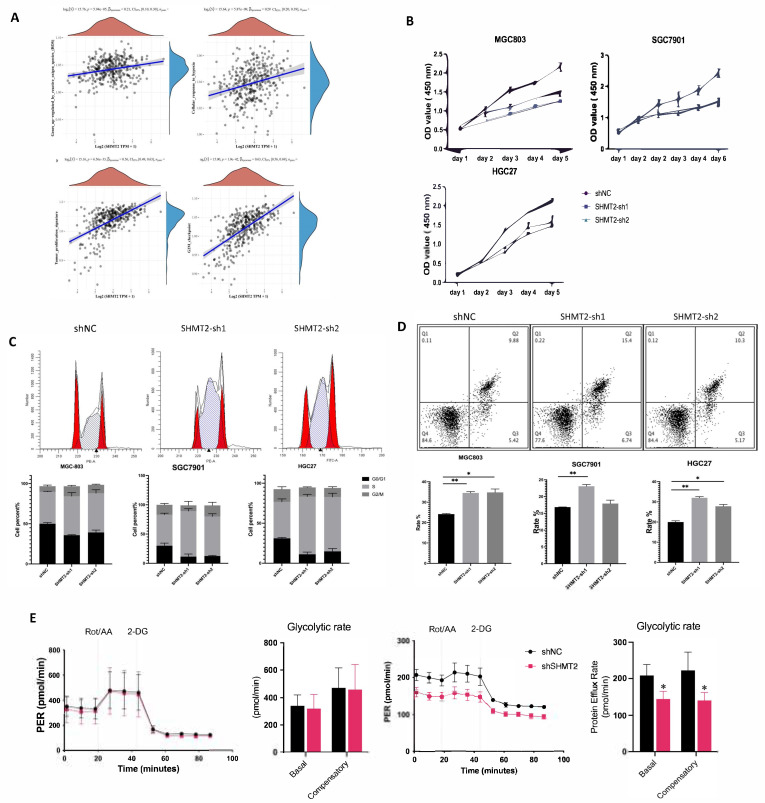
The deletion of SHMT2 inhibited GC cell proliferation, cell cycling, apoptosis, and invasive metastasis under hypoxia. (**A**) Spearman correlation analysis of SHMT2 with cellular response to hypoxia, gene upregulation by reactive oxygen species (ROS), tumor proliferation signature, and G2M checkpoint pathway in TCGA database. The abscissa represented the gene expression distribution, and the ordinate represented the pathway score distribution. The density curve on the right represented the trend in pathway score distribution, the upper density curve represented the trend in gene expression distribution. The value on the top represented the correlation p value, correlation coefficient, and correlation calculation method. (**B**) CCK-8 measured the proliferation of GC cells under normoxic and hypoxic conditions. (**C**,**D**) Effect of SHMT2 knockdown on GC cell cycle and apoptosis under hypoxic conditions. Different colors in the subfigure C indicated different groups, with black being the control group and blue being the knockdown group. (**E**) Effect of SHMT2 knockdown on the mitochondrial glycolysis rate in SGC7901 cells under normoxic and hypoxic conditions. * *p* < 0.05, ** *p* < 0.01.

To investigate SHMT2’s role in GC, we conducted a Spearman correlation analysis between the SHMT2 and pathway scores using the TCGA database. SHMT2 was significantly correlated with multiple signaling pathways, so we focused on the more innovative pathways. We found that, in addition to some metabolic and synthetic pathways, SHMT2 was significantly correlated with the cellular response to hypoxia, gene upregulation by reactive oxygen species (ROS), the tumor proliferation signature, and the G2M checkpoint pathway (Figure 4A). The full correlation analysis of SHMT2 and pathways is available in supplementary data Table S1. Hypoxia is a common and continuous characteristic in many solid tumors and can cause tumor cells to be more likely to invade and metastasize. Thus, studying how tumors behave in a hypoxic environment is essential for understanding how tumors spread and recur [22]. To study SHMT2 deficiency’s impact on GC cells under hypoxic conditions, we used an incubator with low oxygen (1% O_2_) to simulate hypoxia in vitro. We examined the cell proliferation under normoxic and hypoxic conditions using CCK-8 and found that hypoxia significantly reduced cell viability (Figure 4B). The cell cycle analysis revealed that shSHMT2 cells were more significantly inhibited in the S-phase under hypoxic conditions (Figure 4C). The FACS analysis showed that SHMT2 knockdown dramatically increased apoptosis in GC cells (MGC803, SGC7901, and HGC27) under hypoxia compared to normoxia (Figure 4D). We examined the cells’ ability to invade and migrate under hypoxic conditions using clone-formation assays and wound-healing assays, and found that hypoxic conditions further reduced the clone formation, invasion, and migration ability of the shSHMT2 cells compared to the shNC cells (Figures S4 and S5). Since SHMT2 deficiency sensitized GC cells to hypoxia, we examined the glycolytic capacity of SGC7901 under normoxia and hypoxia using seahorse XF and found that the shSHMT2 group had a significantly lower glycolytic capacity than the shNC group at 1% oxygen (Figure 4E). These results suggested that SHMT2 deficiency caused GC cells to be more sensitive to hypoxia and significantly affected the proliferation, cell cycle, apoptosis, clone formation, and invasion and metastasis ability of the GC cells.

### 2.5. SHMT2 Maintained Cell Growth by Sustaining an Oxidative Reduction in Gastric Cancer Cells under Hypoxia

**Figure 5:**
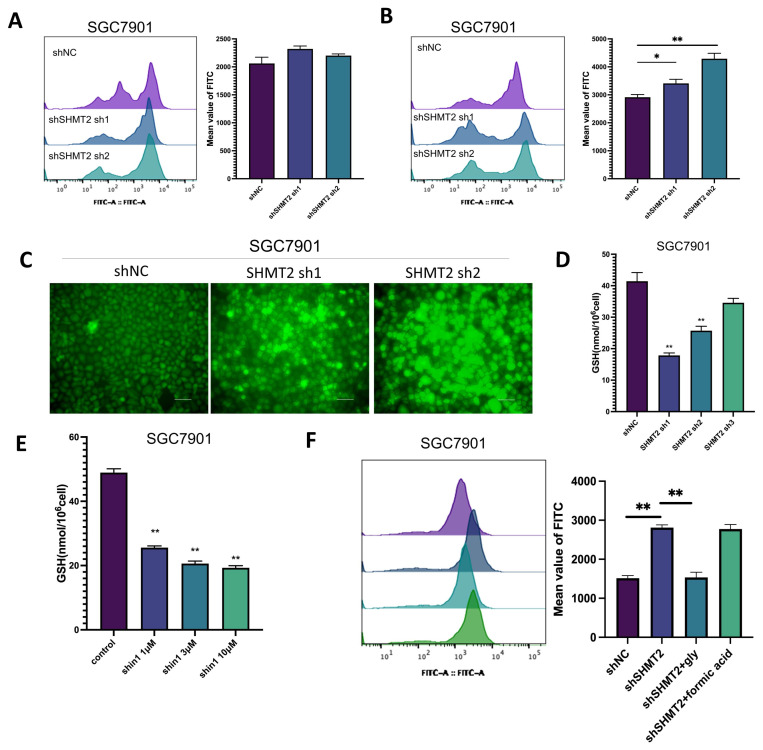
SHMT2 promoted the growth of GC cells by maintaining the redox balance in GC cells under hypoxia. (**A**) Effect of SHMT2 knockdown on ROS levels in SGC7901 cells under normoxic conditions. (**B**) Effect of SHMT2 knockdown on ROS levels in SGC7901 cells under hypoxia conditions. (**C**) Representative pictures of ROS staining of SGC7901 cells under hypoxic conditions with deletion of SHMT2. Scale bar, 100 μm. (**D**) Effect of DOX (300 ng/mL) induced SHMT2 transient deletion on glutathione (GSH) levels in SGC7901 cells under hypoxic conditions. (**E**) The effect of SHIN1 at varying concentrations (1 μM, 3 μM, and 10 μM) on GSH levels in SGC7901 cells during hypoxic conditions. (**F**) Changes in ROS after back supplementation with glycine (1 mM) and formic acid (1 mM) in SGC7901 cells with SHMT2 knockdown under hypoxic conditions. * *p* < 0.05, ** *p* < 0.01.

ROS plays a crucial role in regulating the signal transduction pathways for various cellular functions, and their levels are elevated by hypoxia in many cell types. Initially, the ROS levels were measured in the SGC7901 cells under normoxia between the shSHMT2 and shNC groups, and no significant difference was observed (Figure 5A). However, under hypoxia, the shSHMT2 group showed significantly higher ROS levels than the shNC group (Figure 5B). The ROS levels in the SHMT2 transient knockdown group and the SHIN1 group were also compared to the results of shSHMT2 stabilized cells, which were significantly enhanced under hypoxic conditions (Figures S6 and S7). A schematic illustration of the ROS staining in SGC7901 cells was presented in Figure 5C. The ratio of reduced glutathione (GSH) and oxidized GSH (GSSG) serves as an important indicator of the redox status of the cells. Thus, we also evaluated the levels of GSH in SGC7901 cells (Figure 5D,E). These results indicated that SHMT2 deletion led to cell death by preventing cells from maintaining redox homeostasis under hypoxic conditions. SHMT2 deletion resulted in insufficient glycine synthesis, which could be reversed by supplementing 1 mM glycine. However, the addition of formic acid (1 mM), the end product of OCM, did not reverse the redox imbalance (Figure 5F). Based on the above observations, SHMT2 deficiency caused it to be difficult for GC cells to maintain redox levels in hypoxic conditions.

### 2.6. SHMT2 Contributed to Cell Proliferation under Hypoxia by Maintaining the Redox Balance of Cells via the HIF1a/VEGF/STAT3 Pathway

**Figure 6:**
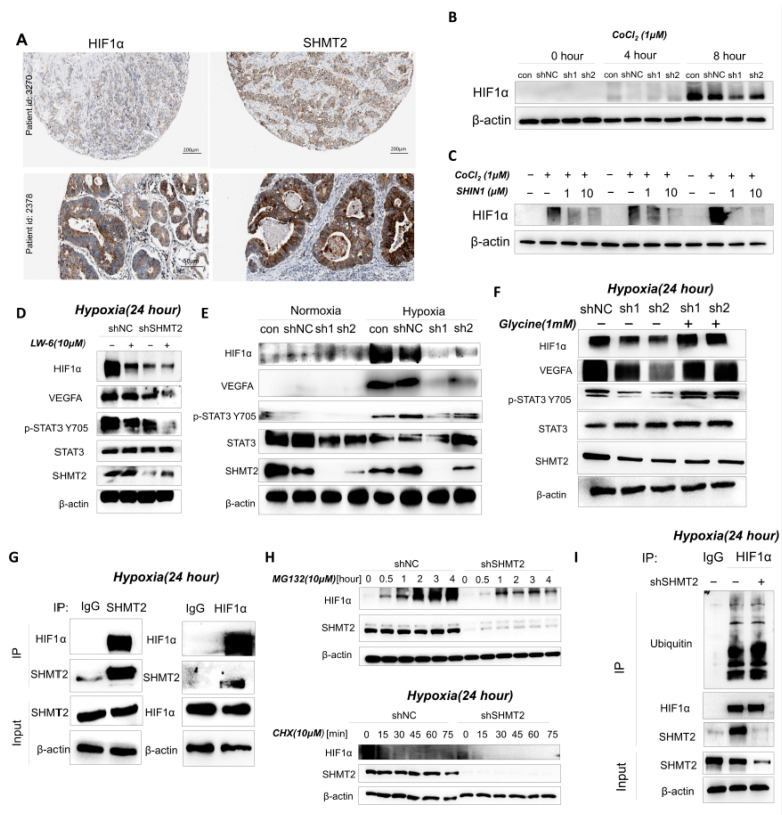
SHMT2 knockdown inhibited the HIF1α/VEGFA/STAT3 signaling pathway. (**A**) Representative IHC staining for SHMT2 and HIF1α in normal gastric tissues and tumor tissues of GC patients from the Human Protein Atlas database. Scale bar: 200 μm and 50 μm. (**B**) Western blot analysis of intracellular HIF1α, VEGFA, and β-actin after stimulation with CoCl_2_ (1 μM) in shSHMT2 SGC7901 cells. (**C**) Western blot analysis of intracellular HIF1α, VEGFA, and β-actin in SGC7901 cells stimulated with CoCl_2_ (1 μM) in the presence of DMSO or SHIN1 for 6 h. (**D**) Western blot analysis of intracellular HIF1α, VEGFA, p-STAT3 (Y705), STAT3, SHMT2, and β-actin under hypoxia with LW-6 (10 μM) in shNC and shSHMT2 group. (**E**) Western blot analysis of HIF1α, VEGFA, p-STAT3 (Y705), STAT3, SHMT2, and β-actin in SGC7901 cells under normoxic and hypoxic conditions after SHMT2 knockdown. (**F**) Western blot analysis of HIF1α, VEGFA, p-STAT3 (Y705), STAT3, SHMT2, and β-actin in shSHMT2 cells after glycine (1 mM) back completion. (**G**) Coimmunoprecipitation assay of SHMT2 and HIF1α in SGC7901 cells after hypoxic stimulation of cells for 24 h. (**H**) Western blot of HIF1α in shNC and shSHMT2 cells treated with MG132 (10 μM) and CHX (10 μM). (**I**) Ubiquitination assay of SHMT2 and HIF1α in shNC and shSHMT2 cells after 24 h of hypoxic stimulation.

Glycolysis plays a significant role in the development, progression, invasion, and metastasis of malignant tumors [23]. HIF1α is a crucial regulator of glucose metabolism in tumor cells and is known to connect numerous important pathways to glycolysis [24]. Additionally, HIF1α is associated with redox homeostasis [25]. The immunohistochemical sections of patients with GC from the Human Protein Atlas database showed that HIF1α and SHMT2 expression were associated and co-expressed (Figure 6A). Based on this clinical relevance, we hypothesized that the phenotype of shSHMT2 cells in hypoxia is associated with HIF1α. We first induced HIF1α using cobalt chloride (CoCl_2_*)*, an agonist of HIF1α, and found that HIF1α was poorly activated in the SGC7901 cells with SHMT2 knockdown (Figure 6B). Similarly, HIF1α was improperly activated in the group treated with CoCl_2_ (10 μM) and SHIN1 in GC cell lines (Figure 6C). We then used LW-6 (10 μM), an inhibitor of HIF1α, to inhibit the SGC7901 cells and compared it with the SHMT2 knockdown group. The results showed that the effect of LW-6 on HIF1α expression was similar to that of the SHMT2 knockdown (Figure 6D). The Western blot analysis showed that HIF1α was not normally activated in the SGC7901 cells with SHMT2 knockdown under normoxia and hypoxia conditions (Figure 6E). VEGF–STAT3, which is directly downstream of HIF1α, is crucial for tumor development [26,27]. The Western blot analysis revealed that the deletion of SHMT2 impaired the HIF1α expression, which consequently impacted the VEGF–STAT3 pathway downstream. Although the deletion of SHMT2 had no effect on the mRNA level of HIF1α, it had a significant effect on the mRNA level of VEGFA, thus suggesting that SHMT2 did not affect the transcription of HIF1α (Figure S8). According to the enzymatic function of SHMT2, the expression level of the HIF1α protein in the shSHMT2 group under hypoxia increased after supplementation with 1 mM glycine. The VEGF and p-STAT3 expressions were also restored (Figure 6G). From a non-enzymatic perspective, we investigated the potential interaction between SHMT2 and HIF1α using a Co-IP assay. Our results indicated that SHMT2 interacted with HIF1α in the GC cells (Figure 6G). We hypothesized that SHMT2 may impact HIF1α’s stability. To test this, we used cycloheximide (CHX), a protein synthesis inhibitor, and MGC132, a proteasome inhibitor, on the shNC and shSHMT2 groups. The Western blot analysis revealed that the deletion of SHMT2 rendered the HIF1α more unstable and prone to degradation (Figure 6H). Additionally, the ubiquitination assay demonstrated that the HIF1α protein level was reduced in the shSHMT2 cells due to an increase in the ubiquitination level (Figure 6I). Collectively, these findings suggested that SHMT2 could affect the HIF1α expression through both enzymatic and non-enzymatic functions. This, in turn, influenced downstream signaling via the VEGF–STAT3 pathway.

### 2.7. SHMT2 Knockdown Suppressed the Tumorigenesis of GC Cells In Vivo

**Figure 7:**
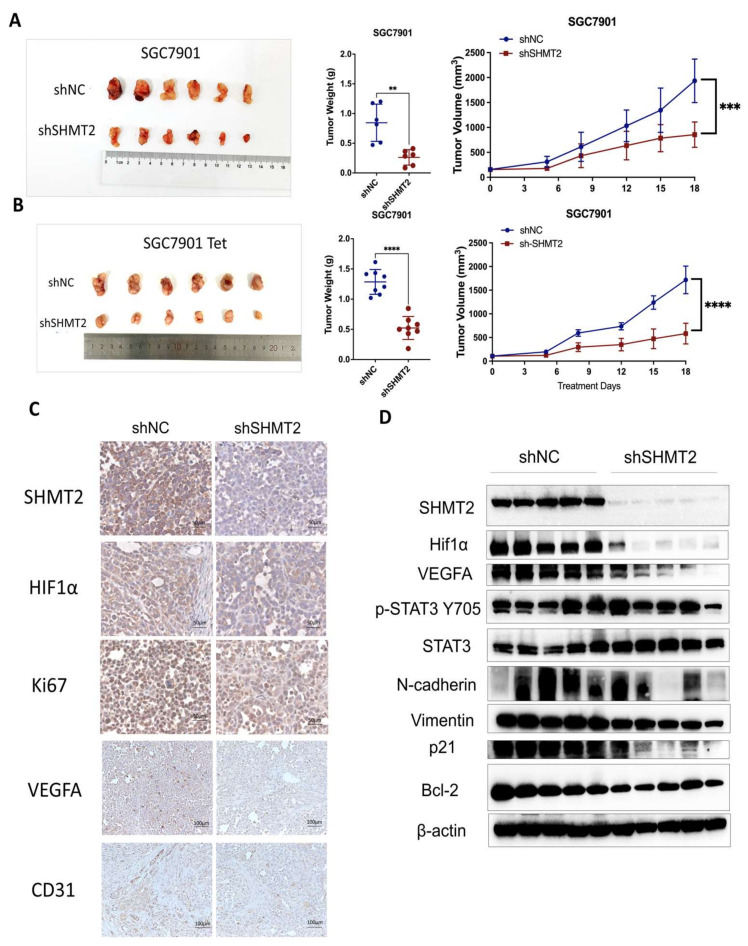
SHMT2 knockdown inhibited GC cell growth in a mice xenograft model in vivo. (**A**) shNC and shSHMT2 cells (2 × 10^6^ cell/mouse) were subcutaneously injected into BALB/c-nude mice (*n* = 6); tumor growth curves and tumor images of experimental endpoints as well as tumor weights. (**B**) shNC and shSHMT2 transient knockdown GC cells (2 × 10^6^ cell/mouse) were subcutaneously injected into BALB/c-nude mice (*n* = 6), and 2 g/L of DOX and 5% of sucrose were added to the daily drinking water of the mice; tumor growth curves and tumor images at the end of the experiment and tumor weights. (**C**) Representative IHC staining of xenograft tumors for SHMT2, Ki67, HIF1α, VEGFA, and CD31. (**D**) Western blotting of SHMT2, HIF1α, VEGFA, p-STAT3 (Y705), STAT3, N-cadherin, vimentin, p21, Bcl-2, and β-actin in xenograft tumor tissues, *n* = 5. ** *p*  <  0.01, *** *p* <  0.001, and **** *p* <  0.0001.

To further confirm SHMT2’s role in gastric carcinogenesis in vivo, we established a subcutaneous transplantation tumor model in nude mice using stable knockdown cell lines of MGC803 and SGC7901. The induced SHMT2 knockdown SGC7901 cells and the control cells. The results showed that the shSHMT2 group had a slower tumor growth rate to the shNC group. The tumor volume and weight were significantly reduced in the shSHMT2 group (Figure 7A,B and Figure S9). The immunohistochemistry results confirmed the SHMT2 knockdown efficiency in the tumor tissue, and the HIF1α staining results were consistent with the in vitro findings, indicating that the HIF1α in the shSHMT2 group was not normally expressed during tumor hypoxia. The downstream gene, VEGFA, was similarly reduced in the shSHMT2 group. The hypoxia was accompanied by vascular neovascularization, and the CD31 expression, a vascular marker, was similarly reduced. The study also revealed that the cell proliferation ratio was significantly reduced in the shSHMT2 SGC7901 group, as evidenced by the Ki-67 staining. (Figure 7C). The Western blot results further confirmed that the expressions of HIF1α, VEGFA, and p-STAT3 were all reduced in the tumor tissue of the shSHMT2 group. Additionally, the expression of EMT-related markers was also reduced in the tumor tissue, as SHMT2 was found to have an effect on EMT in vitro, and the expression of EMT-related markers N-cadherin and vimentin were reduced in the tumor. Additionally, the expression levels of the apoptosis-related markers Bcl-2 and p21 were lower in the experimental than in the control groups (Figure 7D). Collectively, SHMT2 reduction inhibited HIF1α expression in the tumor tissue, reduced tumor malignancy, and slowed tumor cell proliferation in vivo.

## 3. Discussion

Metabolic reprogramming is a key characteristic of cancer [28]. SHMT2, an essential enzyme for cancer metabolic reprogramming, catalyzes the reversible reaction of serine to glycine by transferring the β-carbon from serine to THF [29]. This reaction provides serine as the primary material for the de novo synthesis of purine [30]. In this study, we investigate the biological functions, molecular mechanisms, and clinical implications of SHMT2 in gastric carcinogenesis progression.

SHMT2’s importance in hypoxia has been studied due to its prevalence as a non-physiological level of oxygen stress in most malignancies. Hypoxia is associated with numerous tumor hallmarks, such as cell proliferation, angiogenesis, metabolic reprogramming, and radiation resistance, and can also induce EMT and ROS phenotypes in advanced tumor cells, thus promoting cell invasion and metastasis [31,32, 33]. Studies have shown that SHMT2 can be co-induced by HIF1α and Myc in neuroblastoma tumors, and, in the absence of oxygen, SHMT2 maintains cell growth by balancing the NADPH/NADP^+^ ratios [34]. Additionally, high SHMT2 expression in prostate cancer has been linked to a shift in cell metabolism toward anaerobic metabolism by decreasing serine levels and activating STAT3 transcription [35]. ROS plays a critical role in hypoxia processes, with intracellular levels affecting cell states. Tumor cells have elevated ROS levels due to their high metabolism, receptor signaling, and oxidative enzyme activity [36]. Our study revealed that the deletion of SHMT2 increased the cellular ROS levels, thus leading to cell death.

HIF1α is a key transcription factor adapted to the hypoxic state that upregulates a series of genes related to glycolytic metabolism and contributes to the development of the Warburg effect in tumor cells [37]. Our findings indicated that HIF1α activation enabled normal GC cells to survive in hypoxic environments, while GC cells lacking SHMT2 exhibited increased apoptosis, redox imbalance, cell cycle arrest, and reduced the glycolytic capacity in the absence of oxygen. This failure of HIF1α to properly express itself is associated with the inability of downstream target genes, such as VEGF and GLUT1, to regulate biological functions and promote GC cell development [38]. Our study showed that SHMT2 decreased tumor cell survival by affecting HIF1α expression, which, in turn, altered the downstream VEGF–STAT3 pathway. Concerning how SHMT2 affected HIF1α, we identified SHMT2’s enzymatic and non-enzymatic roles as contributing to its effect.

On one hand, SHMT2’s role as a metabolic enzyme may explain the observed effects. Specifically, SHMT2 deficiency impairs glycine synthesis, which, in turn, leads to an abnormal intracellular redox balance and HIF1α expression under hypoxic conditions. Glycine is a non-essential amino acid in humans and a constituent amino acid of the endogenous-antioxidant-reduced glutathione. It is often exogenously supplemented during times of severe stress and is sometimes considered semi-essential [39]. Research has shown that glycine metabolism is crucial for carcinogenesis and affects tumor cell proliferation rates. Glycine deficiency can slow down rapidly dividing tumor cells [40]. In high-grade gliomas, glycine played a role as an intermediate in nucleotide biosynthesis, which increased with cell proliferation. Elevated glycine levels accelerated tumor growth and progression, causing it to be a potential glioma marker [41]. In this study, we identified a novel role for glycine in GC. Inhibiting SHMT2 hindered the conversion of serine to glycine, thus resulting in an increase in serine levels and a decrease in glycine levels. However, the back supplementation of glycine could restore normal HIF1α expression under hypoxic conditions. Additionally, supplementing with 1 mM glycine lowered ROS levels, increased HIF1α expression, and allowed the cells to survive in a hypoxic environment. This is likely due to glycine’s antioxidant properties, suggesting that tumor cells can use glycine to survive in hypoxic environments. This investigation provided evidence that glycine back supplementation can restore normal HIF1α expression in hypoxic environments, thus revealing a previously unknown role for glycine in cancer biology.

On the other hand, the observed effects may also be related to the non-enzymatic function of SHMT2. SHMT2 reportedly has multiple non-enzymatic functions, including its ability to interact with β-catenin and inhibit ubiquitination-mediated β-catenin degradation, ultimately promoting the proliferation and metastasis of colorectal cancer cells [42]. Under normoxia, HIF1α binds to VHL proteins and is degraded by ubiquitination. However, under hypoxia conditions, HIF1α does not attach to VHL but instead directly enters the nucleus, where it binds to specific genomes and activates and regulates the function of numerous genes involved in oxygen supply and transport [43]. Through our experiments, we discovered that SHMT2 could interact with HIF1α, and that the depletion of SHMT2 led to a significant decrease in the stable HIF1α protein levels in GC cells. Furthermore, we found that HIF1α ubiquitination was increased in cells with SHMT2 depletion, suggesting that SHMT2 enhanced the HIF1α protein stability under hypoxic conditions, facilitating its expression. This study is the first to demonstrate that SHMT2 can interact with HIF1α and affect the HIF1α ubiquitination to influence its expression.

This study’s limitations are, firstly, that, although we demonstrated the correlation between SHMT2 and HIF1α and explained how SHMT2’s effects through both its metabolic and non-metabolic enzyme functions, the exact mechanism has not been fully explored, so further studies are necessary. Secondly, while we observed that SHMT2 depletion affected the invasive metastasis of GC cells in vitro, a valid in vivo model of GC metastasis is necessary to confirm these findings.

## 4. Materials and Methods

### 4.1. Analyses Based on Public Databases

All the data for GC patients were downloaded from the TCGA database (https://portal.gdc.cancer.gov (accessed on 23th June 2022)) that collates RNAseq data on TCGA-STAD (gastric cancer). The prognostic data were obtained from an article published in Cell [44].

### 4.2. Cell Culture and Reagents

The GC cell lines (MGC803, MKN45, HGC27, SGC7901, and AGS), normal gastric cells GES1, and HEK293T were obtained from the China Infrastructure of Cell Line Resources (Beijing, China). The GC cells were cultured in RPMI-1640 medium while the GES1 and HEK293T were cultured in DMEM medium. All the media were complemented with 10% fetal bovine serum (FBS, Hyclone, Logan, UT, USA), 100 units/mL penicillin, and 100 μg/mL streptomycin (Living, Beijing, China). All the cells were maintained in a humidified incubator containing 5% CO_2_ at 37 °C. The cobalt chloride (#232696) and glycine (#V900144) were purchased from Sigma-Aldrich (St. Louis, MO, USA). The formic acid was purchased from Thermo Fisher Scientific (#85178, Waltham, MA, USA). The doxycycline was purchased from Topscience (#T1687, Shanghai, China).

### 4.3. Lentiviral Packaging and Virus Infection

Standard protocols were followed to generate viruses, and packaging lentiviruses were used for constructing the stable transgenic cells. The lentiviral packaging plasmids psPAX2 and pMD2.G and shRNA were used to generate the lentiviral particles in the HEK293T cells. The supernatants containing virus particles were collected at 48 h and 72 h post-transfection and concentrated using a lentivirus precipitation solution (#FV101-01, Transgen Biotech, Beijing, China). The concentrated virus particles and polybrene (1:1000, #C0351, Beyotime, Shanghai, China) were used to infect the GC cells grown in 6-well plates. The stable shRNA-expressing cells were selected using puromycin (0.25 mg/mL, #ant-pr-1, InvivoGen, San Diego, CA, USA) for 3–5 days. The shRNA sequences were as follows:

shRNA#1: 5′-CCGGAGAGTTGTGGACTTTAT-3′.

shRNA#2: 5′-CAACCTCACGACCGGATCAT-3′.

shRNA#3: 5′-GCAACGGGTGGAGCAGTTTGC-3′.

### 4.4. Western Blot Analysis

The total protein was extracted using RIPA lysis buffer (#R0010, Solarbio, Beijing, China) containing protease inhibitor and protein phosphatase inhibitor. The protein concentrations were detected using the BCA protein quantification kit. Equal amounts of protein (20 μg) were loaded onto 10% SDS-PAGE and then transferred onto PVDF membranes (#IPVH00010, Millipore, Billerica, MA, USA). All the membranes were then blocked with 5% BSA for 1 h at room temperature and incubated with a primary antibody overnight at 4 °C. The next day, the membranes were incubated with HRP-coupled secondary antibodies (1:1000, #2301, ZS-Bio, Beijing, China) for 2 h at room temperature. The protein signal was visualized using the ECL assay kit (#180-5001, Tanon, Shanghai, China). β-actin was used as an internal reference. The primary antibodies are as follows: SHMT2 (#33443), N-cadherin (#13116), E-cadherin (#14472), HIF1α (#36169), p-STAT3 (Y705) (#9145), STAT3 (#9139), vimentin (#5741), Bcl2 (#15071), p21 (#2947) (1:1000, Cell Signaling Technology, Danvers, MA, USA), VEGFA (#19003) (1:1000, Proteintech, Wuhan, China), and β-actin (1:2000, #TA-09, ZS-Bio).

### 4.5. Real-Time Quantitative PCR

Briefly, the total mRNA was extracted using RNA extraction reagents (#RN001, Esunbio, Shanghai, China) according to the previous steps, and the concentration of RNA was detected using Biotek (Biotek, Winooski, VT, USA). Additionally, reverse transcription was performed using the quantitative reverse transcription kit StepOnePlus™ RT-PCR System (#AT-341, Transgen Biotech). Real-time quantitative PCR was performed using SYBR Green qPCR Master Mix (#1198, Yeasen, Shanghai, China). The target genes were normalized using GAPDH as a control, and the results were calculated using the 2-ΔΔCt method.

The primer sequences were as follows:

SHMT2-F: 5′-CCCTTCTGCAACCTCACGAC-3′.

SHMT2-R: 5′-TGAGCTTATAGGGCATAGACTCG-3′.

HIF1α-F: 5′-GAACGTCGAAAAGAAAAGTCTCG-3′.

HIF1α-R: 5′- CCTTATCAAGATGCGAACTCACA-3′.

VEGFA-F: 5′-AGGGCAGAATCATCACGAAGT-3′.

VEGFA-R: 5′- AGGGTCTCGATTGGATGGCA-3′.

### 4.6. CCK-8 Cell Viability Assay

The cells were inoculated into 96-well plates (2 × 10^4^ cells/well); 10 μL of CCK-8 reagent (#C0005, Topscience) was added to each well and incubated for 2 h on days 0, 1, 2, 3, 4, and 5. The absorbance (OD) at 450 nm was used to obtain the cell viability.

### 4.7. Cell Cycle and Cell Apoptosis

The cell cycle analysis was performed using 75% cold ethanol to fix the cells overnight at 4 °C. The next day, the cells were stained using propidium iodide (PI)/RNase staining buffer (#550825, BD Biosciences, San Jose, CA, USA). The cells were then analyzed using FACS Calibur (BD Biosciences). The cell cycle distribution was quantified using ModFit LT software.

For apoptosis, the cells were stained using Annexin V-FITC/PI binding buffer (#C1062L, Beyotime). The cells were then analyzed using FACS Calibur (BD Biosciences). The percentage of the apoptotic cells was quantified using FlowJo software 10.4.

### 4.8. EdU Assay

The EdU assays were performed using the EdU Assay Kit (#C0078S, Beyotime). The cells were inoculated into 6-well plates (2 × 10^4^ cells/well), incubated with EdU (10 μM) for 2 h, and fixed in 4% formaldehyde (#P1110, Solarbio) for 30 min. The cells were then permeabilized with 0.3% TritonX-100 (#T8200, Solarbio) for 10 min, allowing the cells to react with the Apollo reaction mixture for 30 min. The nuclear staining of the cells was performed with Hoechst 33342 (#62249, Invitrogen, Carlsbad, CA, USA,) for 30 min and observed under a microscope.

### 4.9. Wound-Healing Assay and Transwell Assay

For the wound-healing assay, apply a 24-well plate and culture the cells to 90% confluence. A 1 mL sterile pipette tip was used to scratch the cell surface. The images of the wound closure were collected at 0 h and 24 h using a microscope.

For the transwell migration assay, 200 μL of FBS-free medium containing 1 × 10^5^ cells was added to the upper chamber of the transwell chamber. The lower chamber was filled with 600 μL of the 10% FBS medium. After 24–48 h of incubation, the cells were fixed and stained with methanol and 0.25% crystalline violet (#C8470, Solarbio). The stained cells were counted using a microscope and the average number of cells in three random fields was calculated as the result.

### 4.10. Clonogenic Assay and Soft Agar Colony Formation Assay

The cells were inoculated at a rate of 1 × 10^3^ cells per well in 6-well plates in 2 mL of medium. The medium was changed every three days with fresh medium and the cells were allowed to grow for 10 days. The colonies were fixed in 4% formaldehyde, stained using crystalline violet, and then imaged for counting.

For the soft agar cloning experiments, 1.2% agarose gel was mixed with 2 × RPMI1640 in a 1:1 ratio and added to a 6-well plate and allowed to solidify. The 0.7% agarose gel was mixed with 2 × RPMI1640 in a 1:1 ratio and then mixed with cells, added to the well plates, and incubated in an incubator for 3 weeks. The culture medium was replenished every three days. Photographs were taken and counted using an inverted microscope, and the average number of cells in three random fields was calculated as the result.

### 4.11. Seahorse XF Glycolysis Assay

The glycolysis rate was evaluated using the Seahorse XFe24 Analyzer (Agilent Technologies, Santa Clara, CA, USA). The medium was replaced with 500 μL pH 7.4 ± 0.1 bicarbonate-free DMEM supplemented with 1 mmol/L sodium pyruvate, 2 mmol/L glutamine, and 10 mmol/L glucose according to the manufacturer’s instructions. In total, 5 × 10^4^ GC cells were seeded per well in special 24-well plates and cultured overnight. Then, the well plates were incubated for 1 h at 37 °C in a CO_2_-free incubator. The ECAR was measured using the Seahorse XF Glycolytic Stress Test Kit (Agilent Technologies). Under all conditions, 10 mmol/L glucose, 0.5 μM oligomycin, and 50 mmol/L 2-deoxyglucose (2-DG) were used at final concentrations.

### 4.12. ROS Assay and GSH Assay

The GC cells were inoculated in 6-well plates at 3 × 10^5^ cells per well, and, then, the cells were stained using 10 μM DCFDA (#CA1410, Solarbio) at 37 °C for 30 min. After staining, the cells were washed twice and the ROS signal was measured using flow cytometry.

For the glutathione assay, no less than 5 million cells were collected, repeatedly freeze–thawed 2–3 times in liquid nitrogen and a 37 °C water bath, centrifuged at 8000× *g* for 10 min, and the supernatant was collected for the assay. The levels of reduced glutathione were determined using the Glutathione Fluorescence Assay Kit (#BC1175, Solarbio) according to the manufacturer’s instructions. A standard curve of the GSH was established to determine the free GSH concentration in the samples. The results were expressed as μmol per mg soluble proteins.

### 4.13. Animal Experiments

Six-week-old BALB/c nude mice were obtained from HuafuKang (Beijing, China). These animals were housed at 24 °C under specific pathogen-free conditions with a 12-h diurnal cycle. They had free access to a gamma-irradiated laboratory rodent diet. All the experiments were performed following the relevant guidelines and regulations of the Institute of Materia Medica (IMM), Chinese Academy of Medical Science (CAMS), and Peking Union Medical College (PUMC). The animal protocols for these experiments were approved by the Animal Experimental Center, IMM, CAMS, and PUMC (0000-8699). The mice were randomly divided into six per group, and the stably transfected GC cells (5 × 10^6^/mouse) were inoculated in 200 μL of Matrigel suspension into the mice’s left lower axillas. Doxycycline (2 g/L) and sucrose (5%) were in the mice’s drinking water for daily consumption for 2 weeks. The tumor dimensions were measured every three days using vernier calipers, and the tumor volume was estimated as tumor volume = length × width^2^ /2, where the length indicates the maximum tumor diameter and the width indicates the diameter perpendicular to the length. The tumors were collected and used for the histological analysis. All the animal experiments were repeated at least twice and similar results were obtained.

### 4.14. Immunohistochemical Analysis

The tissues were fixed in a 4% paraformaldehyde solution and embedded in paraffin. The tissue sections were dewaxed in xylene, hydrated in graded alcohol solution, and heated (100 °C) in citric acid solution (pH 6.0) for antigen repair. For single immunostaining, the endogenous peroxidase in the tissue was blocked using endogenous peroxidase blocking solution, and 5% BSA was used to block the tissue’s nonspecific proteins. The sections were incubated overnight at 4 °C with the indicated antibodies, followed by incubation with the corresponding horseradish peroxidase-coupled secondary antibodies (#PV9001, Beyotime). Finally, 3,3′-diaminobenzidine (#PV6000D, Beyotime) was used to detect these labeled antibodies and the nuclei were stained using hematoxylin. The primary antibodies were as follows: SHMT2 (1:100, #33443, Cell Signaling Technolog), HIF1α (1:100, #36169, Cell Signaling Technology), VEGFA (1:100, #19003, Proteintech), KI67 (1:50, #5032, PTM Bio, Hangzhou, China), and CD31 (1:50, #ab28364, Abcam, Cambridge, MA, USA).

### 4.15. Statistical Analysis

All the experiments were performed using three separate time replicates. All the statistical analyses were performed using the GraphPad Prism 7 software and SPSS 20.0. The differences between the two groups were compared using Student’s *t*-test. One- or two-way ANOVAs were used to compare multiple groups. The results are shown as mean ± standard deviation (SD). The overall survival was compared using the log–rank test and expressed as Kaplan–Meier survival curves. To assess the correlation between SHMT2 and hypoxia marker expression in the TCGA data, we used a Pearson correlation analysis. *p* < 0.05 was set as statistically significant.

## 5. Conclusions

In conclusion, this study highlights SHMT2’s clinical and prognostic significance in GC. Our findings suggest that SHMT2 promotes GC progression by modulating the HIF1α/VEGF/STAT3 signaling pathway. To overcome the potential compensatory mechanisms associated with metabolic enzyme knockdown, we employed multiple models of SHMT2 knockdown. Our results indicate that SHMT2 is a promising target for diagnosing and treating GC.

## Data Availability

The data sets used and/or analyzed during the current study can be obtained from the corresponding author if reasonably requested.

## Supplementary Materials

The following supporting information can be downloaded at: https://www.mdpi.com/article/10.3390/ijms24087150/s1, Figure S1: Effect of SHIN1 on proliferation of gastric cancer cells; Figure S2: Effect of SHIN1 on cell cycle of gastric cancer; Figure S3: Effect of SHIN1 on cell apoptosis of gastric cancer; Figure S4: Effect of SHMT2 deficiency on wound healing capacity under hypoxia; Figure S5: Effect of SHMT2 deficiency on clonogenesis under hypoxia; Figure S6: Effect of induced transient knockdown of SHMT2 on ROS levels in GC cells under hypoxia; Figure S7: Effect of SHIN1 on ROS levels in GC cells under hypoxia.; Figure S8: HIF1α and VEGFA mRNA levels in GC cells; Figure S9: SHMT2 knockdown inhibited MGC803 cells growth in a mice xenograft model in vivo; Table S1: Correlation analysis between SHMT2 and pathways in TCGA database.
