# Supplementary material for: SHMT2 Promotes Gastric Cancer Development through Regulation of HIF1α/VEGF/STAT3 Signaling
Source: Int J Mol Sci. 2023 Apr 12;24(8):7150. doi: 10.3390/ijms24087150 (PMC10138966; doi:10.3390/ijms24087150)
Supplement: Supplementary file 1 [file ijms-24-07150-s001.zip › supplymental data.pdf]

Figure S2-1

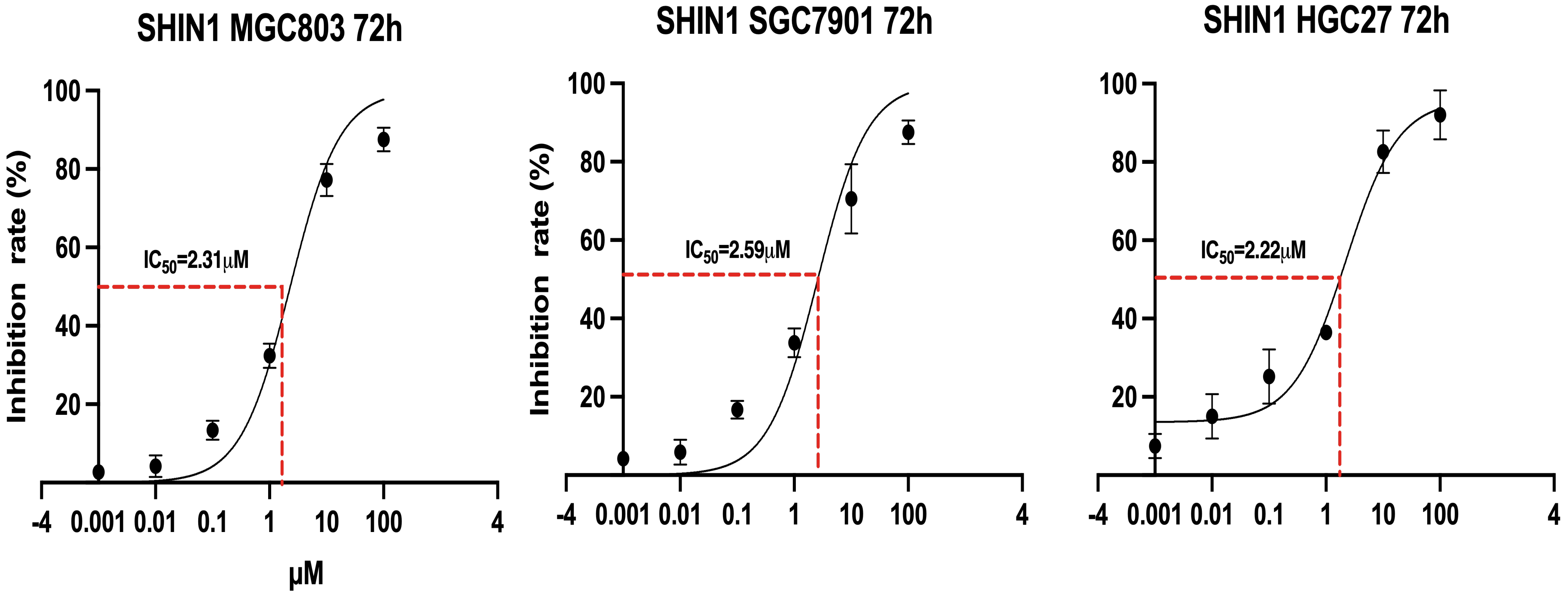
SHIN1 MGC803 72h
SHIN1 SGC7901 72h
SHIN1 HGC27 72h
Inhibition rate (%)
IC50=2.31μM
IC50=2.59μM
IC50=2.22μM
100
80
60
40
20
-4
0.001
0.01
0.1
1
10
100
4
μM

Figure S2-2

SGC7901

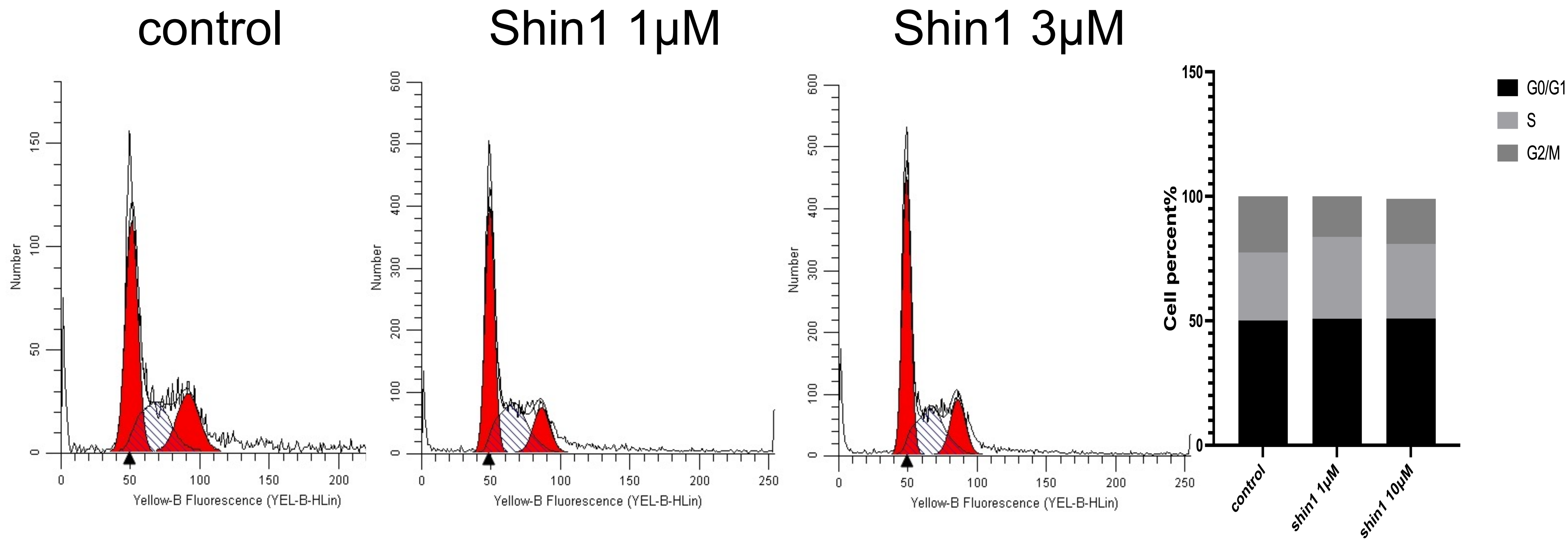

Figure S2-3

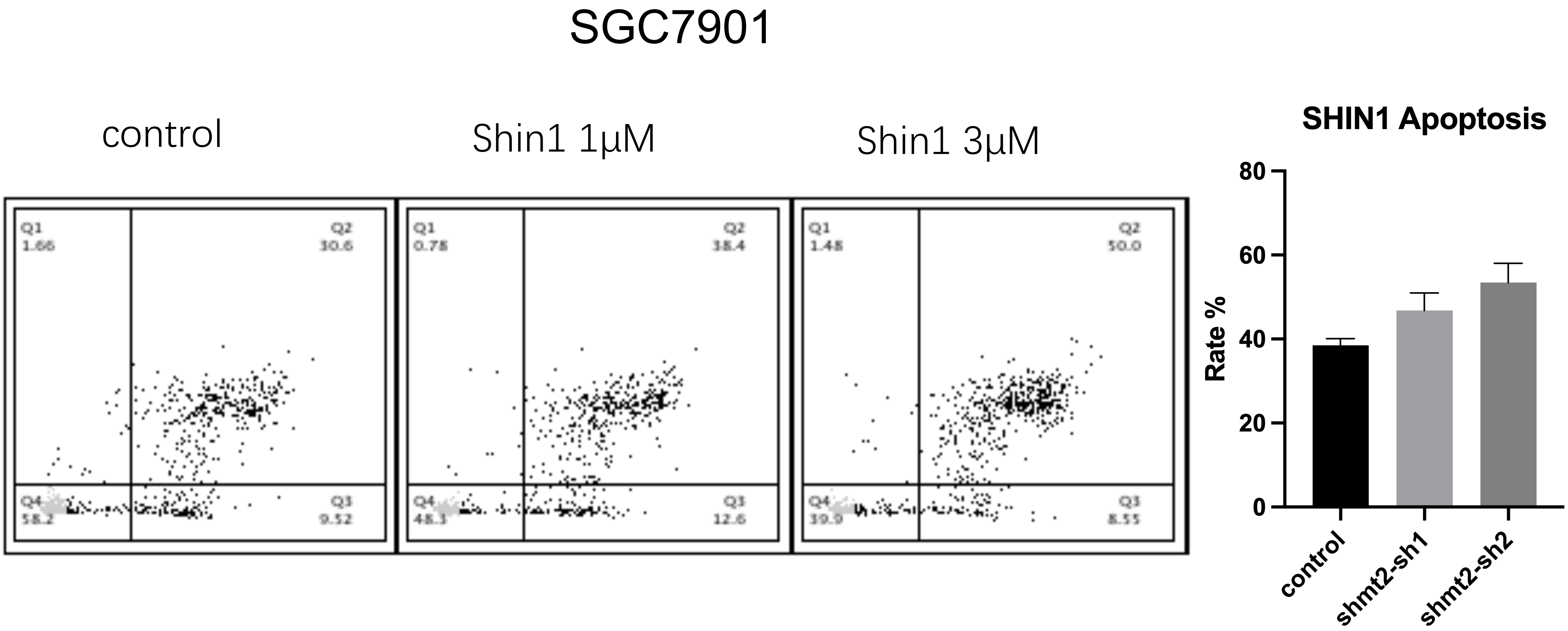
SGC7901
control
Shin1 1μM
Shin1 3μM
Q1
1.66
Q2
30.6
Q4
58.2
Q3
9.52
Q1
0.78
Q2
38.4
Q4
48.3
Q3
12.6
Q1
1.48
Q2
50.0
Q4
39.9
Q3
8.55
SHIN1 Apoptosis
Rate %
80
60
40
20
0
control
shmt2-sh1
shmt2-sh2

Figure S4-1

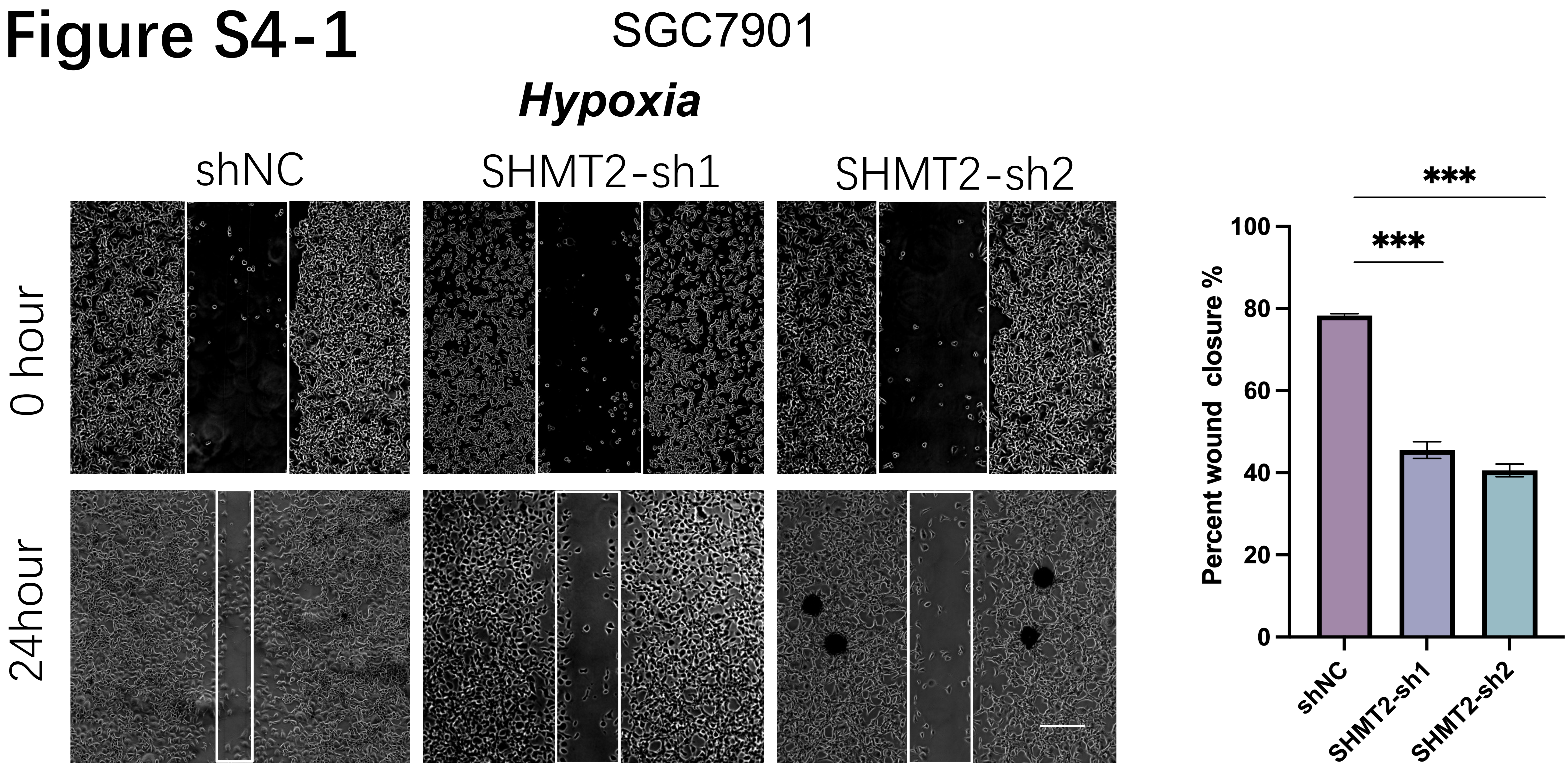
SGC7901
Hypoxia
shNC
SHMT2-sh1
SHMT2-sh2
0 hour
24hour
***
***
Percent wound closure %
100
80
60
40
20
0
shNC
SHMT2-sh1
SHMT2-sh2

Figure S4-2

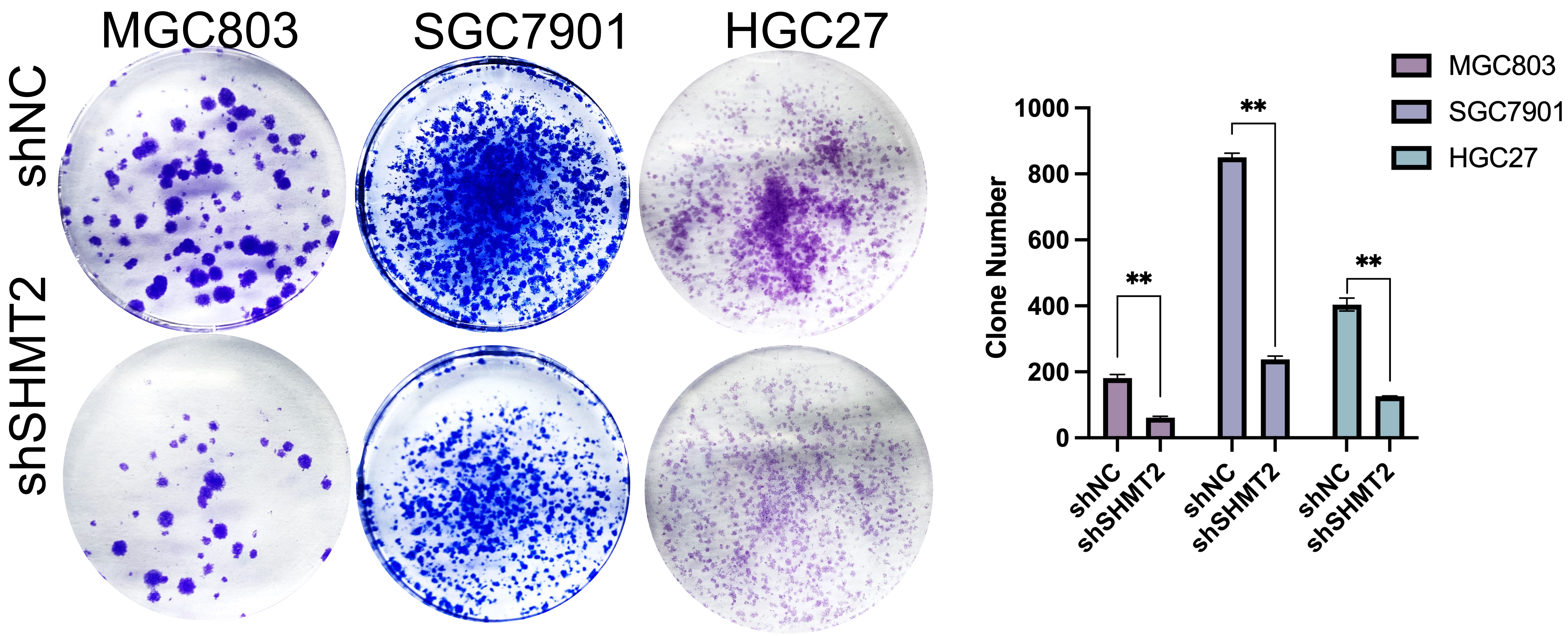
MGC803
SGC7901
HGC27
shNC
shSHMT2
Clone Number
1000
800
600
400
200
0
**
**
**
MGC803
SGC7901
HGC27
shNC
shSHMT2
shNC
shSHMT2
shNC
shSHMT2

Figure S5-1

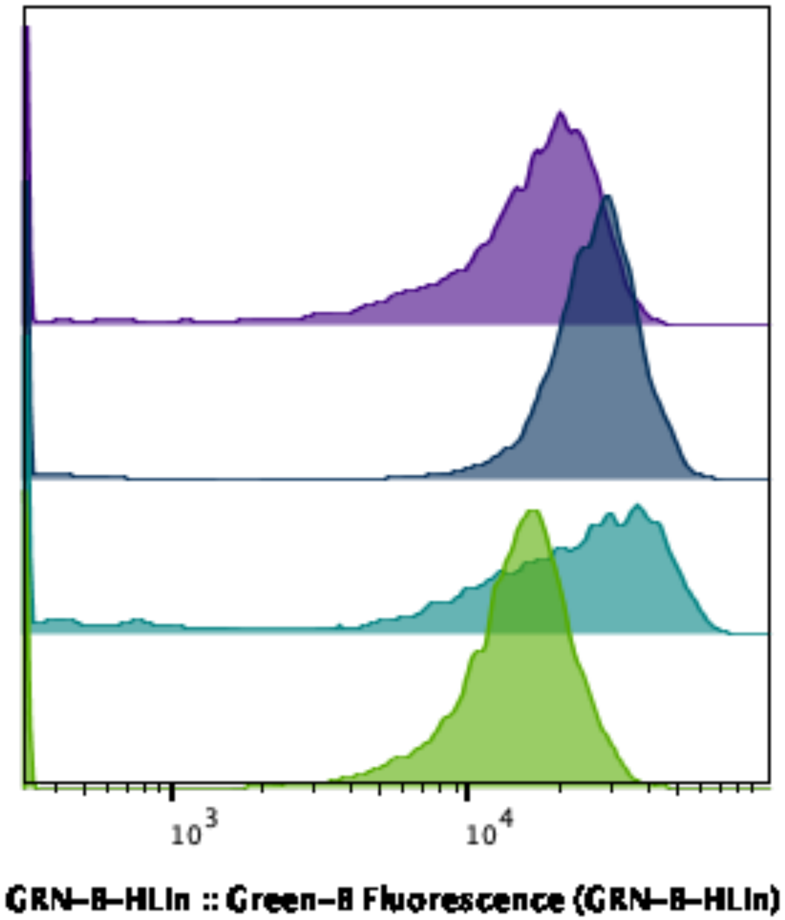

10^3
10^4
GRN-B-HLin :: Green-B Fluorescence (GRN-B-HLin)

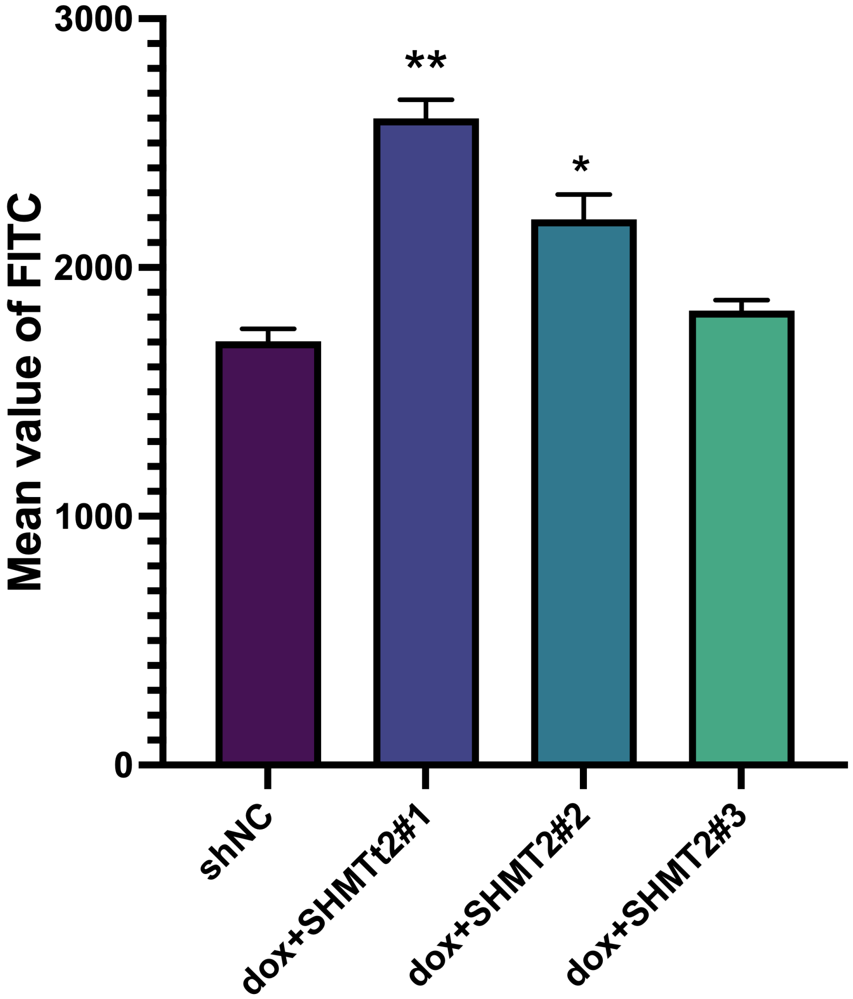

Mean value of FITC
3000
2000
1000
0
**
*
shNC
dox+SHMTt2#1
dox+SHMT2#2
dox+SHMT2#3

Figure S5-2

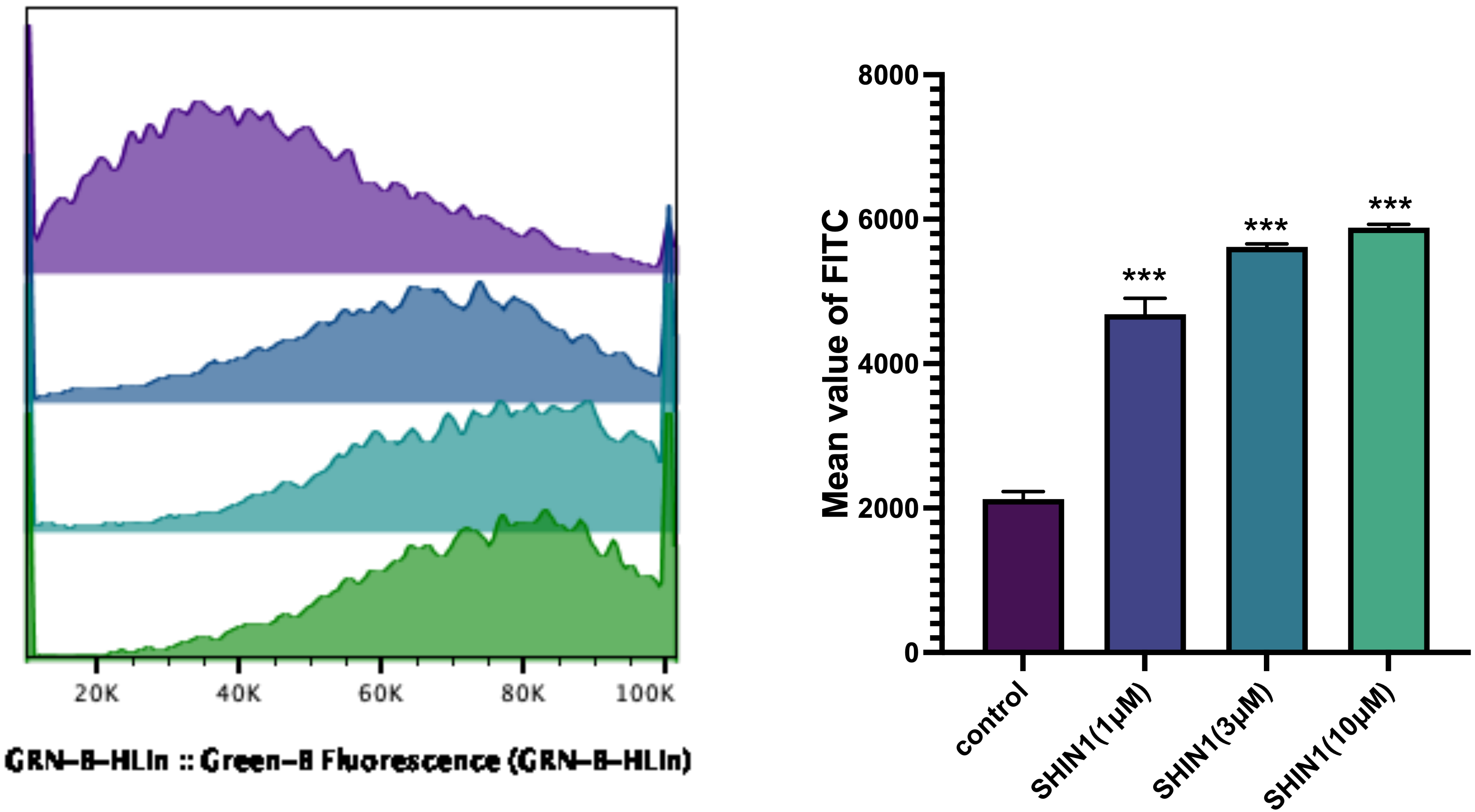
20K
40K
60K
80K
100K
GRN-B-HLin :: Green-B Fluorescence (GRN-B-HLin)
8000
6000
4000
2000
0
Mean value of FITC
***
***
***
control
SHIN1(1μM)
SHIN1(3μM)
SHIN1(10μM)

Figure S6-1

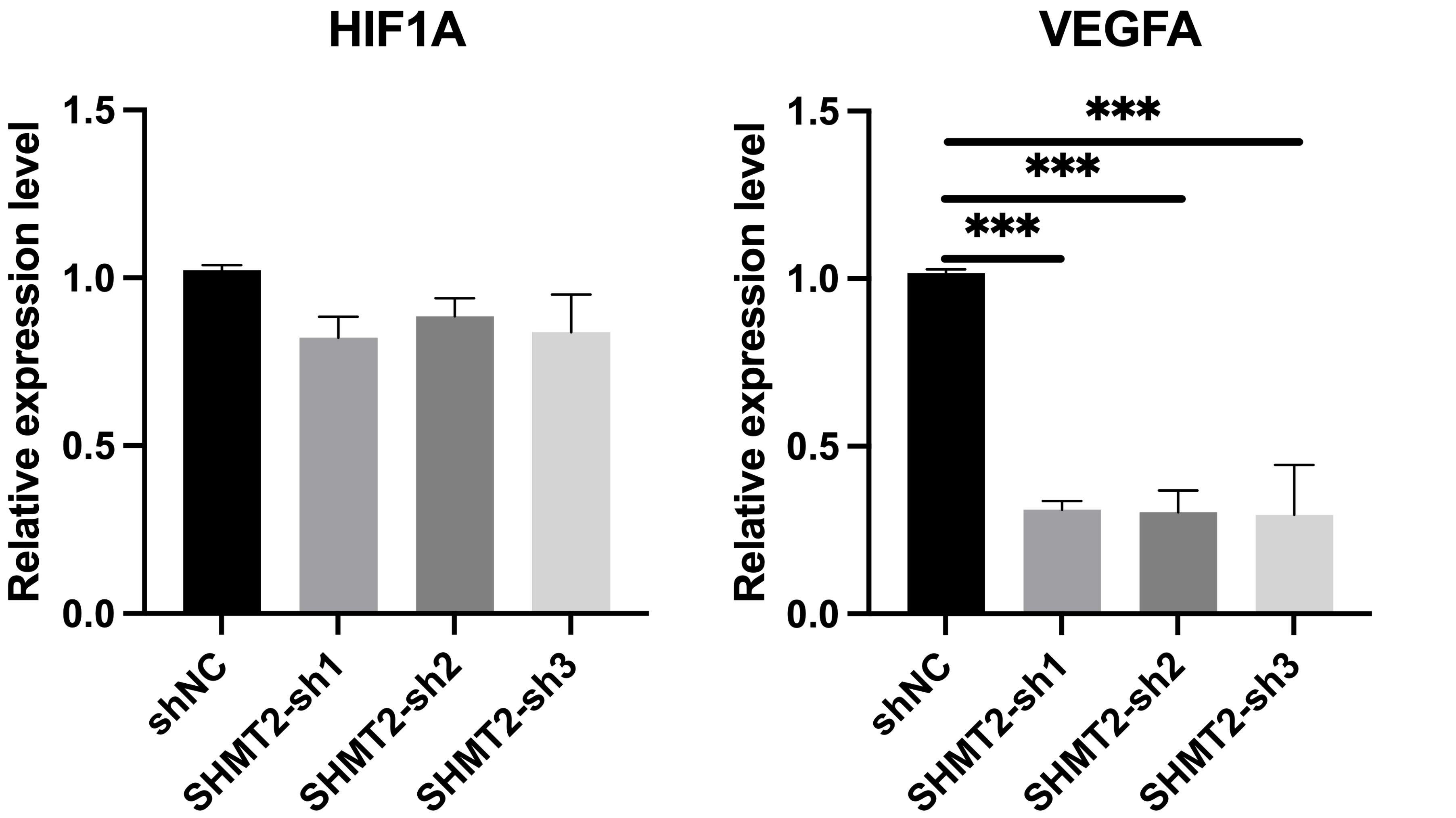
HIF1A
Relative expression level
1.5
1.0
0.5
0.0
shNC
SHMT2-sh1
SHMT2-sh2
SHMT2-sh3
VEGFA
Relative expression level
1.5
1.0
0.5
0.0
***
***
***
shNC
SHMT2-sh1
SHMT2-sh2
SHMT2-sh3

Figure S7-1

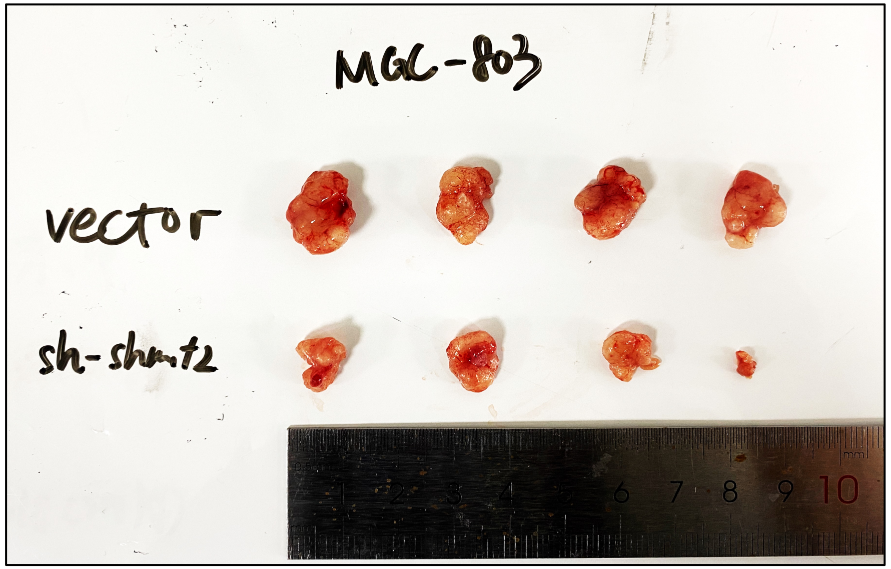

MGC-803
vector
sh-shmt2

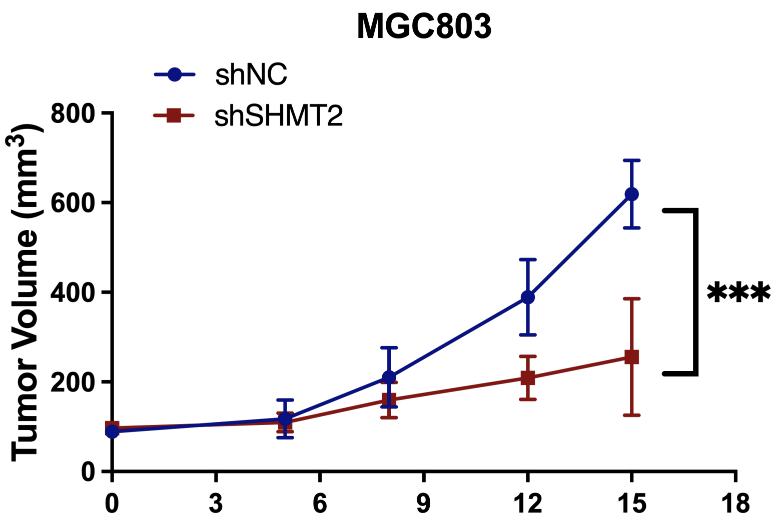

MGC803
shNC
shSHMT2
Tumor Volume (mm^3)
800
600
400
200
0
0
3
6
9
12
15
18
***

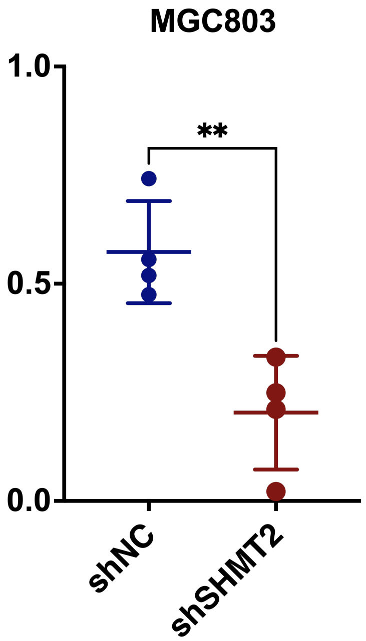

MGC803
1.0
0.5
0.0
**
shNC
shSHMT2
